# Supplementary material for: Vibrio cholerae requires oxidative respiration through the bd-I and cbb3 oxidases for intestinal proliferation
Source: PLoS Pathog. 2022 May 2;18(5):e1010102. doi: 10.1371/journal.ppat.1010102 (PMC9109917; doi:10.1371/journal.ppat.1010102)
Supplement: S1 Methods — (PDF) [file ppat.1010102.s001.pdf]

Supplemental Methods

Hydrogen Peroxide (H_2O_2) Sensitivity Assay

Bacterial strains were grown ON in 4ml LB streptomycin (100µg/ml) broth. After ~16h of growth, cultures were diluted to 1.0 OD600. In a 96-well plate all wells were filled with 100µl LB broth and in the second row 100µl of LB 20mM H_2O_2 (Fisher Chemical) was added and serially diluted to the last row where 100µl was removed leaving a volume of 100µl in all wells. In a 2ml tube, 1.7µl of fresh LB was inoculated 1:500 (2:1000 / 3.4µl) with the 1.0 OD600 cultures. Tubes were vortexed and inoculated media was distributed in duplicate columns for each strain and included blank LB control lanes. 96-well plates were grown at 37°C 210rpm on a plate shaker for 20h. After 20h, the OD600 was read, and percent bacterial growth was determined in comparison to the first row which lacks added H_2O_2.

AKI Virulence Inducing Conditions

Standard AKI Conditions

Bacterial strains were struck on LB streptomycin (100µg/ml) plates and used to inoculate 4ml LB media. After ~16h bacterial strains were diluted to a 1.0 OD600. These cultures were then used to inoculate 40ml prewarmed AKI media 1:5000 (8µl) which were then inverted and set to grow static at 37°C for 4h. After the 4h timepoint, 19ml of culture was transferred to sterile 125ml Erlenmeyer flasks and grown at 37°C, 210rpm, for an additional hour to the experiment 5h timepoint. At both the 4h and 5h timepoint, samples for cholera toxin and TcpA quantification were taken. At the 4h and 5h timepoints, 20ml and 10ml of culture, respectively, were centrifuged at 4000rpm, 4°C, for 10min. For cholera toxin analysis, 1ml of supernatant was removed and stored at -80°C. For TcpA quantification, the bacterial pellet was resuspended in

Resuspension Buffer (50mM Tris-HCl [pH 7.4], 50mM EDTA [pH 8.0]) and transferred to a 1.7ml Eppendorf tube where an equal volume of Lysis Buffer (1% SDS, 10mM Tris-HCl [pH 7.4]) was added. The bacterial solution was vortexed for 10s, boiled for 10min, and then stored at -80°C.

Anaerobic AKI Conditions

Bacterial strains were struck on LB streptomycin (100µg/ml) plates and used to inoculate 4ml LB deoxygenated media. After ~16h bacterial strains were concentrated and resuspended to a 1.0 OD600 anaerobically. These cultures were then used to inoculate 40ml prewarmed deoxygenated AKI media 1:5000 (8µl) which were then inverted and set to grow static at 37°C for 8h. After 8h, tubes were removed from the anaerobic chamber and 20ml volume centrifuged at 4000rpm, 4°C, for 10min. For cholera toxin analysis, 1ml of supernatant was removed and stored at -80°C. For TcpA quantification, the bacterial pellet was resuspended in Resuspension Buffer (50mM Tris-HCl [pH 7.4], 50mM EDTA [pH 8.0]) and transferred to a 1.7ml Eppendorf tube where an equal volume of Lysis Buffer (1% SDS, 10mM Tris-HCl [pH 7.4]) was added. The bacterial solution was vortexed for 10s, boiled for 10min, and then stored at -80°C.

V. cholerae Terminal Oxidase Strain M9 Glucose Growth Curves

Bacterial strains were struck on LB streptomycin (100µg/ml) and used to inoculate 4ml LB deoxygenated media. After ~16h bacterial strains were concentrated to a 1.0 OD600 anaerobically. These cultures were used to inoculate 700µl M9 0.2% Glucose 1:250 (2.8µl), vortexed, and aliquoted in triplicate 200µl volumes in a 96-well plate. Optical density was recorded every hour for the duration of the growth curve. Deoxygenated M9 0.2% Glucose was used for anaerobic growth curves and benchtop media used for aerobic growth curves.

Oxidase TMPD Test Strips

Bacterial strains were tested for functional cytochrome c oxidase *cbb*$_3$ using a rapid test DrySlide containing $N_1N_1N'_1N'$-tetramethyl-*p*-phenylene-diamine dihydrochloride (Wurster's blue; TMPD). Strains were grown on LB Str100 agar media 18-24h and cell collections spotted onto DrySlide using a wooden applicator. Color was allowed to develop, and images taken.

Whole Genome Sequencing Submission and Analysis

DNA of select *V. cholerae* strains was submitted to the Microbial Genome Sequencing Center (MiGS) for whole genome sequencing following sample submission guidelines. Reads were trimmed for low quality base calls and aligned to NCBI reference genome ASM1308507v1, El Tor C6706 *V. cholerae* using Geneious software to generate genome assemblies. Genomes were aligned using Mauve and genomic polymorphisms recorded.

TcpA Western Protein Electrophoresis and Immunodetection

Bacterial stains were grown under standard and anaerobic AKI conditions (49). Briefly, for standard AKI conditions, 40ml of AKI media was inoculated (1:5000) with a 1.0 OD_{600} bacterial cell suspension and incubated at 37°C static for 4hr and switched to shaking for one additional hour at which point sample cell pellets were harvested. For anaerobic AKI conditions, 40ml of deoxygenated AKI media was inoculated (1:5000) with a 1.0 OD_{600} bacterial cell suspension and incubated at 37°C static for 8hr in anaerobic conditions at which point sample cell pellets were harvested. Bacterial cell pellets were first resuspended in resuspension buffer (50mM Tris-HCl [pH 7.4], 50mM EDTA [pH 8.0]), lysed with addition of a lysis buffer (1% SDS,

10mM Tris-HCl [pH 7.4]), and boiled 10 minutes. After cell lysis, the total protein concentration of each sample was measured via Bradford assay (Sigma Aldrich). Samples were subsequently diluted to a final concentration of 0.5 µg total protein/µl. Samples were loaded on an SDS page gel which contained 12.5% acrylamide and run at 120 volts for 1.5 hours. Proteins were transferred to a nitrocellulose membrane using a semi dry electroblotter (Fisher Scientific) overnight at 35 mA. Membranes were blocked with 15 ml of blocking buffer (5% non-fat milk, 2% bovine serum albumin, 0.5% Tween-20, in Tris-buffered saline) for 1 hour at room temperature. α-TcpA antibodies were diluted 1:100,000 in 5% non-fat milk and incubated with the membranes for 1 hour at room temperature. Membranes were washed three times for five minutes with Tris-buffered saline. Goat anti-Rabbit IgG-HRP antibodies (Sigma Aldrich) were diluted 1:2,000 in 5% non-fat milk in Tris-buffered saline and incubated as before. Membranes were washed three times for five minutes with Tris-buffered saline, and then incubated with SuperSignal HRP Chemiluminescence substrate (Thermo Fisher) for five minutes at room temperature. Membranes were then imaged with an Amersham Imager 600.

Wild Type Aerobic, Microaerobic, and Anaerobic RNA Isolation and Real-Time Quantitative PCR (RT-qPCR)

Modified Pfaffl Method Relative Expression Quantification Calculation

$$RQ = \frac{\frac{X_0}{R_0}}{\frac{Co_0}{R_0}} = \frac{\frac{X_T}{R_T}\ x\ \frac{{E_R}^{Ct,R}}{{E_x}^{Ct,x}}}{\frac{Co_T}{R_T}\ x\ \frac{{E_R}^{Ct,R}}{{E_{Co}}^{Ct,Co}}}$$

RQ = relative fold change. X_0 = initial number of experimental target molecules. R_0 = initial number of reference molecules. Co_0 = initial number of comparator target molecules. X_T =

number of experimental target molecules to reach SYBR C_t threshold. R_T = number of reference target molecules to reach SYBR C_t threshold. Co_T = number of comparator target molecules to reach SYBR C_t threshold. E_X = PCR amplification efficiency of experimental target. E_R = PCR amplification efficiency of reference. E_{Co} = PCR amplification efficiency of comparator target. $C_{t,X}$ = SYBR cycle threshold value for experimental target. $C_{t,R}$ = SYBR cycle threshold value for reference. $C_{t,Co}$ = SYBR cycle threshold value for comparator target. Number of DNA molecules required to reach SYBR C_t threshold and PCR efficiency values were determined by generating a standard curve for each target using known quantities of purified genomic DNA as template for all primer pairs in the qRT-PCR reaction.
