# Supplementary figures and images for: Vibrio cholerae requires oxidative respiration through the bd-I and cbb3 oxidases for intestinal proliferation
Source: PLoS Pathog. 2022 May 2;18(5):e1010102. doi: 10.1371/journal.ppat.1010102 (PMC9109917; doi:10.1371/journal.ppat.1010102)

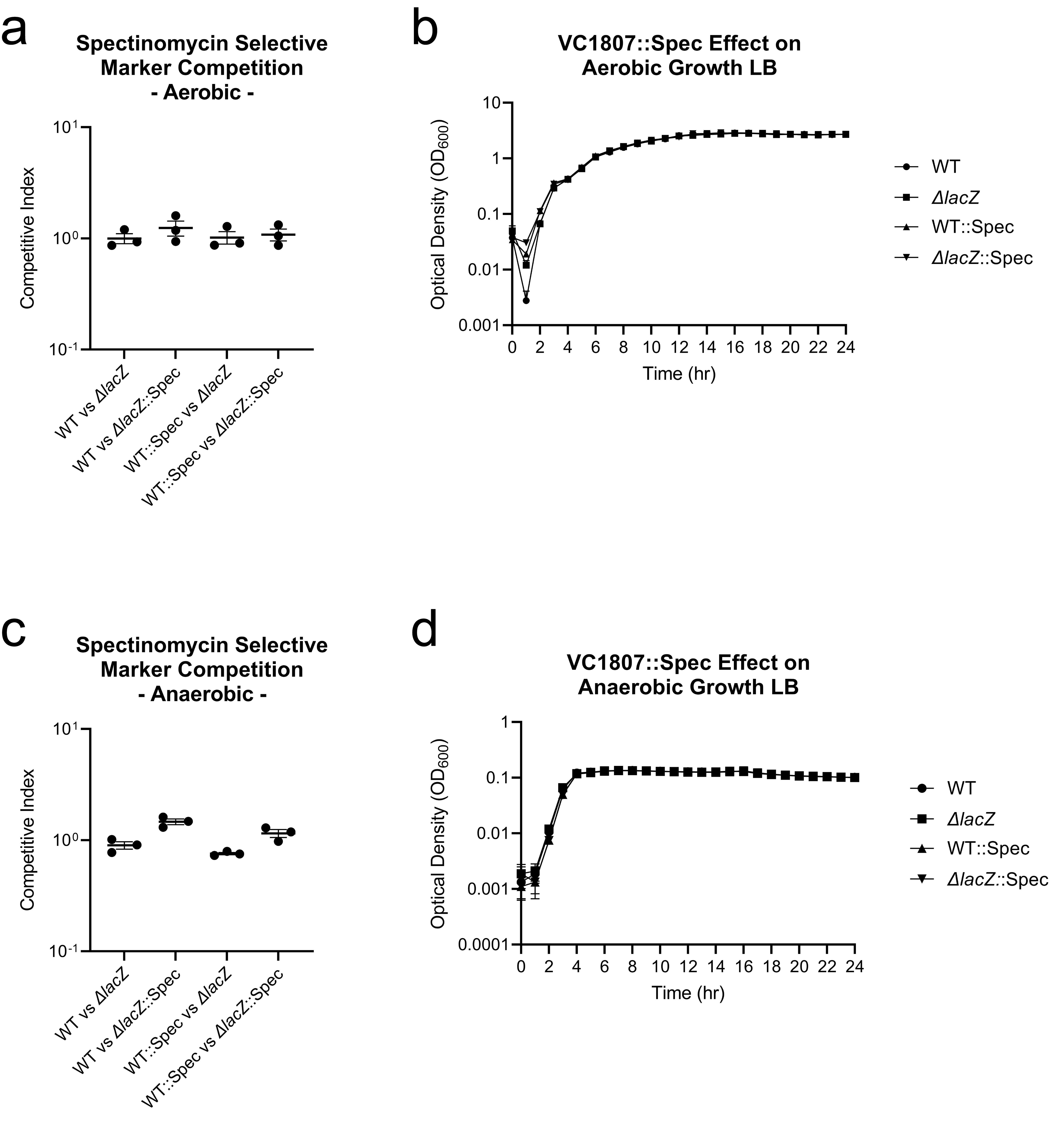

Supplement: S1 Fig — Comparison of wild type and ΔlacZ C6706 V. cholerae strains with and without MuGENT spectinomycin selective marker in pseudogene VC1807. (a) Aerobic in vitro competition assay after 20h. (b) Aerobic growth curve assay. (c) Anaerobic in vitro competition assay after 20h. (d) Anaerobic growth curve assay. All assays were performed in LB media. Bars in in vitro competitions represent the arithmetic mean where error bars represent the standard error of the mean. Growth curves are an average of three biological replicates where error bars represent the standard error of the mean. (TIF) [file ppat.1010102.s002.tif]

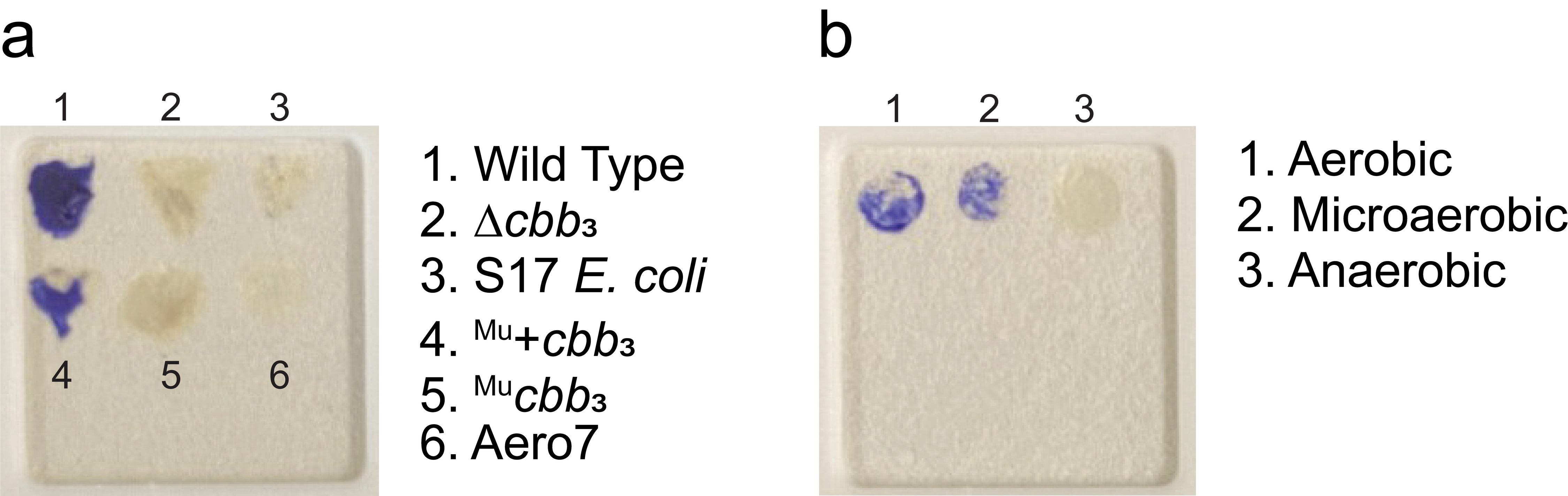

Supplement: S2 Fig — V. cholerae cultures were grown on LB agar plates and spotted onto a rapid test DrySlide containing N1N1N’1N’-tetramethyl-p-phenylene-diamine dihydrochloride (Wurster’s blue; TMPD) that turns blue when reduced by cytochrome c oxidases. This colorimetric detection assay indicates active cytochrome c oxidases but does not provide quantitative measurements of complex activity. (a) V. cholerae cbb3 mutant strain spots and E. coli (cytochrome c deficient) control. (b) Wild type V. cholerae grown in aerobic, microaerobic, and anaerobic conditions. (TIF) [file ppat.1010102.s003.tif]

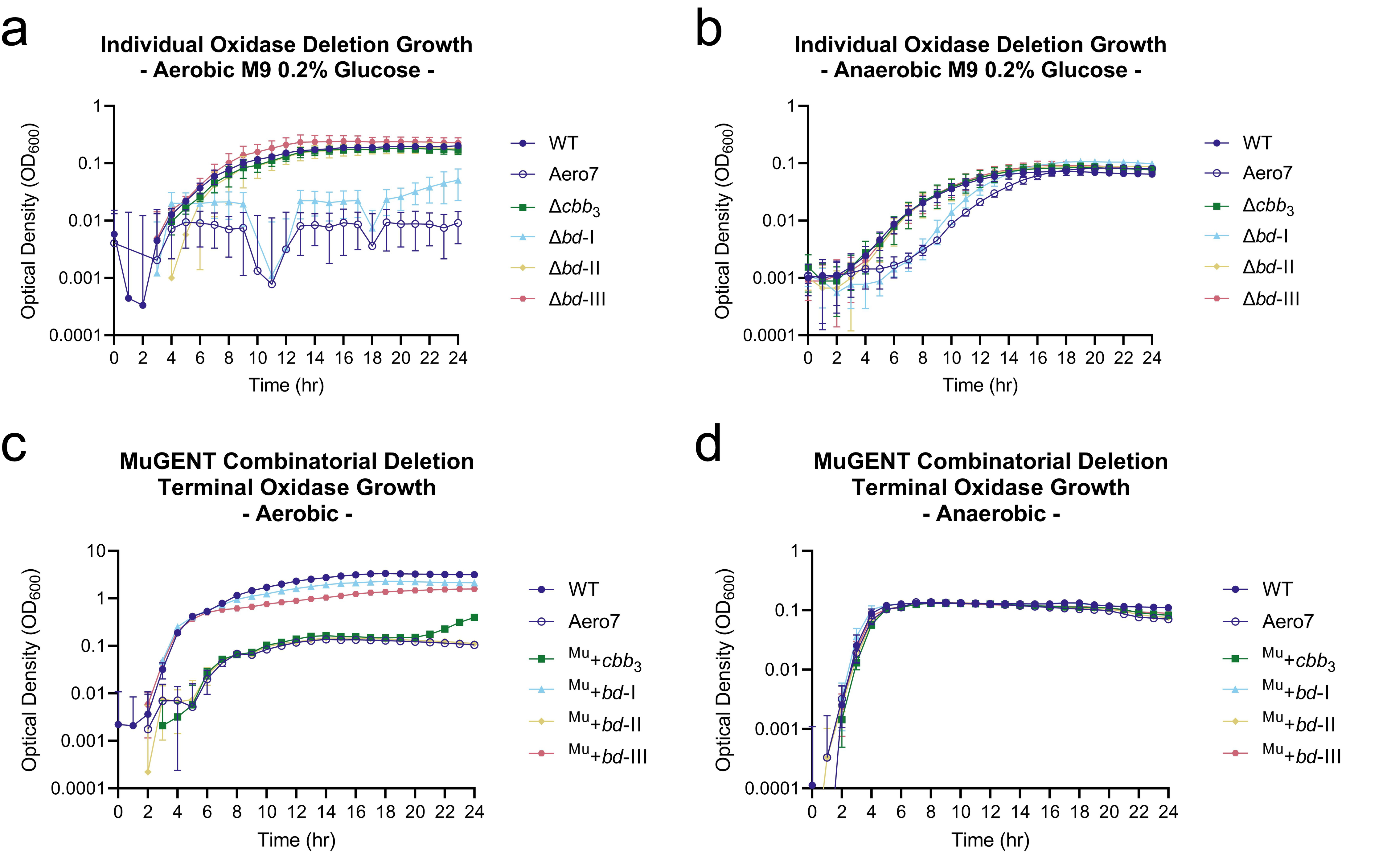

Supplement: S3 Fig — (a-b) Single terminal oxidase deletion mutants grown in M9 0.2% D-glucose, aerobically and anaerobically, respectively. Inoculums for all growth experiments were prepared in anaerobic conditions. (c-d) Combinatorial terminal oxidase deletion mutant growth in LB. Inoculums were prepared anaerobically and subsequently grown in aerobic and anaerobic conditions, respectively. Triple deletion mutant strains have a ‘+’ with an oxidase name (e.g. +cbb3), indicating the sole remaining oxidase, with the other three oxidases disrupted by mutation. Growth curves are an average of three biological replicates where error bars represent the standard error of the mean. (TIF) [file ppat.1010102.s004.tif]

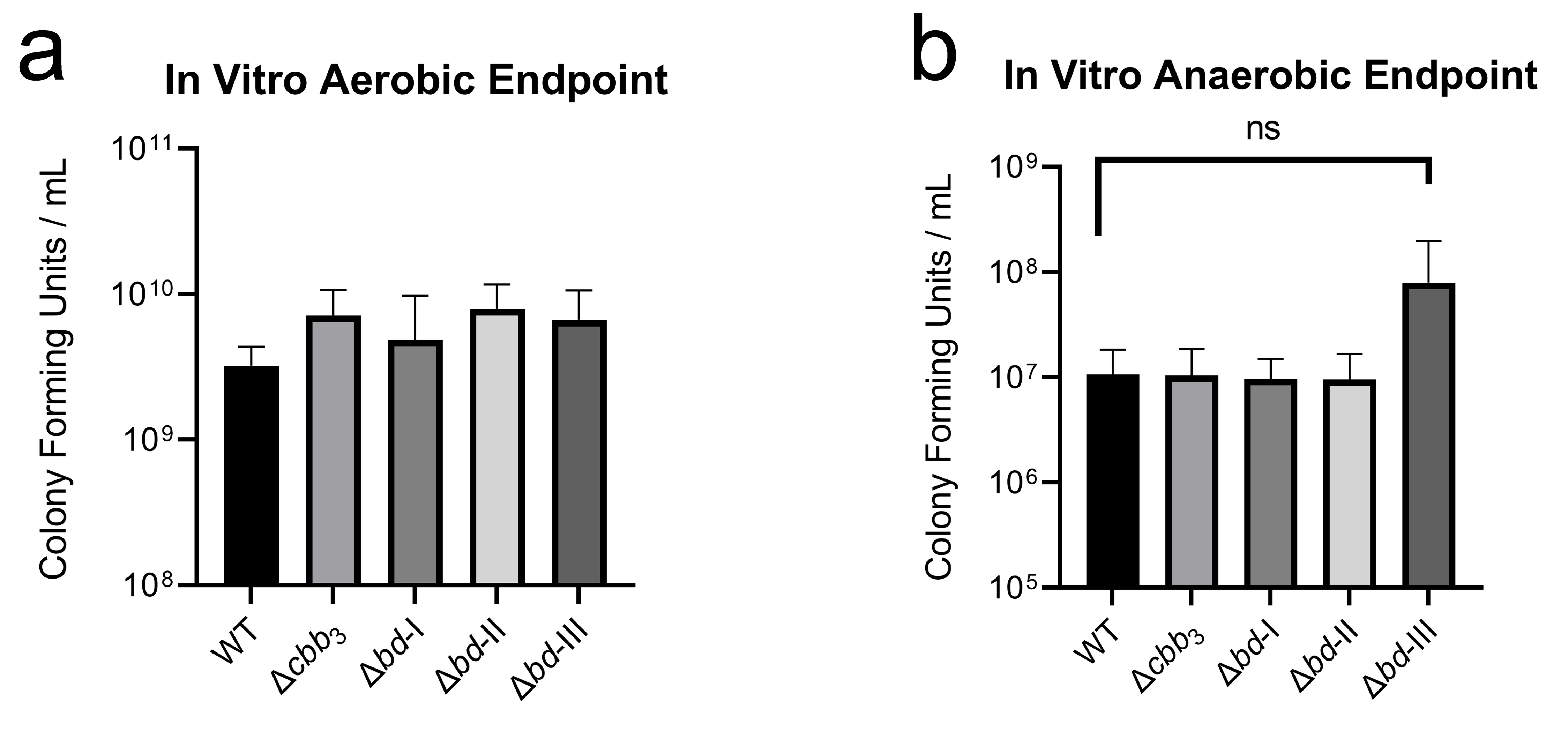

Supplement: S4 Fig — V. cholerae individual deletion strain endpoint CFU in LB media at 20h. CFU endpoints were determined in (a) aerobic and (b) anaerobic growth conditions. CFU’s reported are an average of three biological replicates where error bars represent the standard error of the mean. (TIF) [file ppat.1010102.s005.tif]

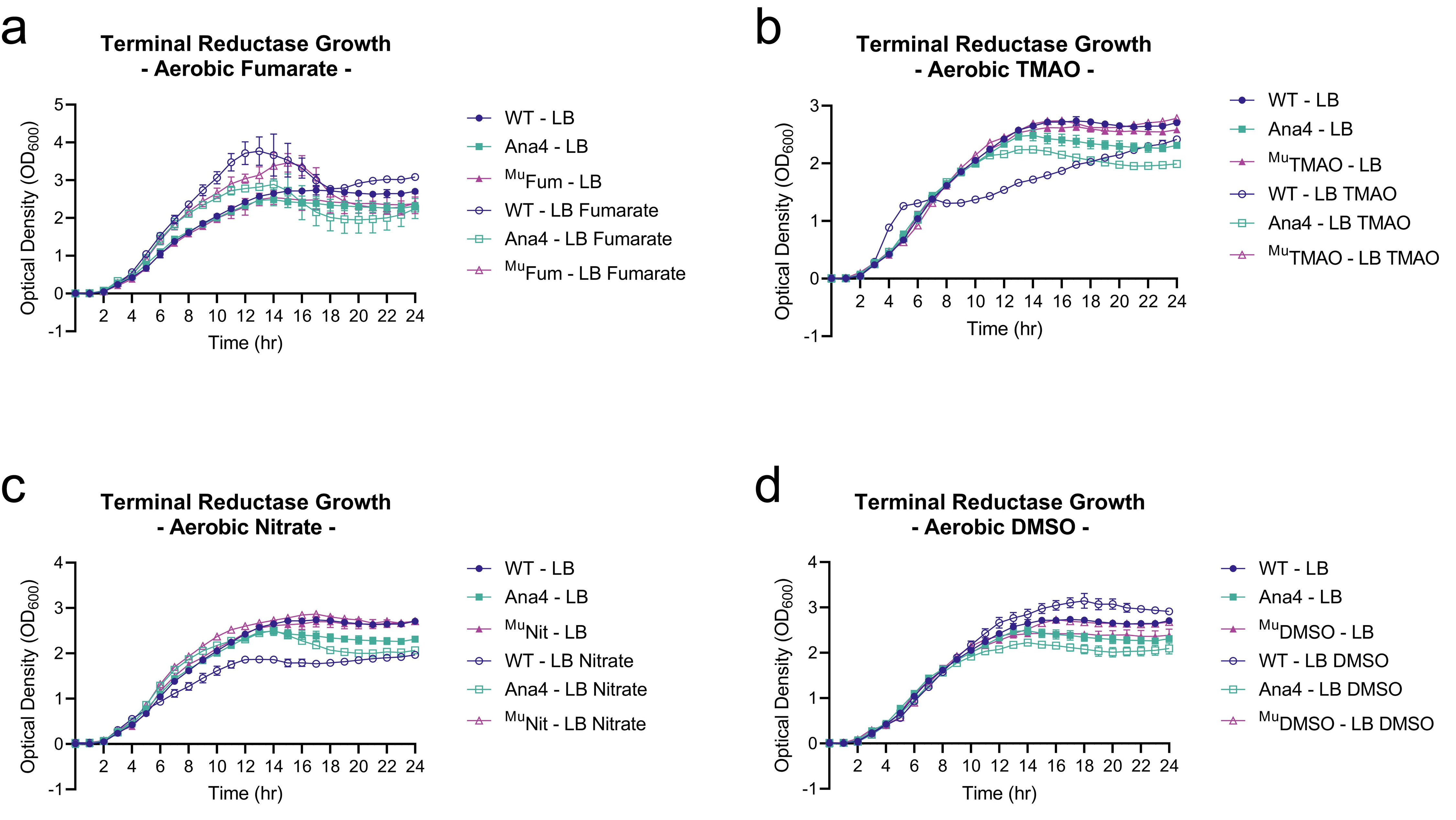

Supplement: S5 Fig — V. cholerae terminal reductase aerobic growth characteristics in LB in the presence and absence of alternative electron acceptors (a) 50mM fumarate, (b) 50mM trimethylamine-N-oxide (TMAO), (c) 50mM nitrate, and (d) 50mM dimethyl sulfoxide (DMSO). Inoculums were prepared aerobically. Closed symbols indicate LB growth media lacked an alternative electron acceptor whereas open symbols indicate LB growth media was supplemented with a given alternative electron acceptor. Growth curves are an average of three biological replicates where error bars represent the standard error of the mean. (TIF) [file ppat.1010102.s006.tif]

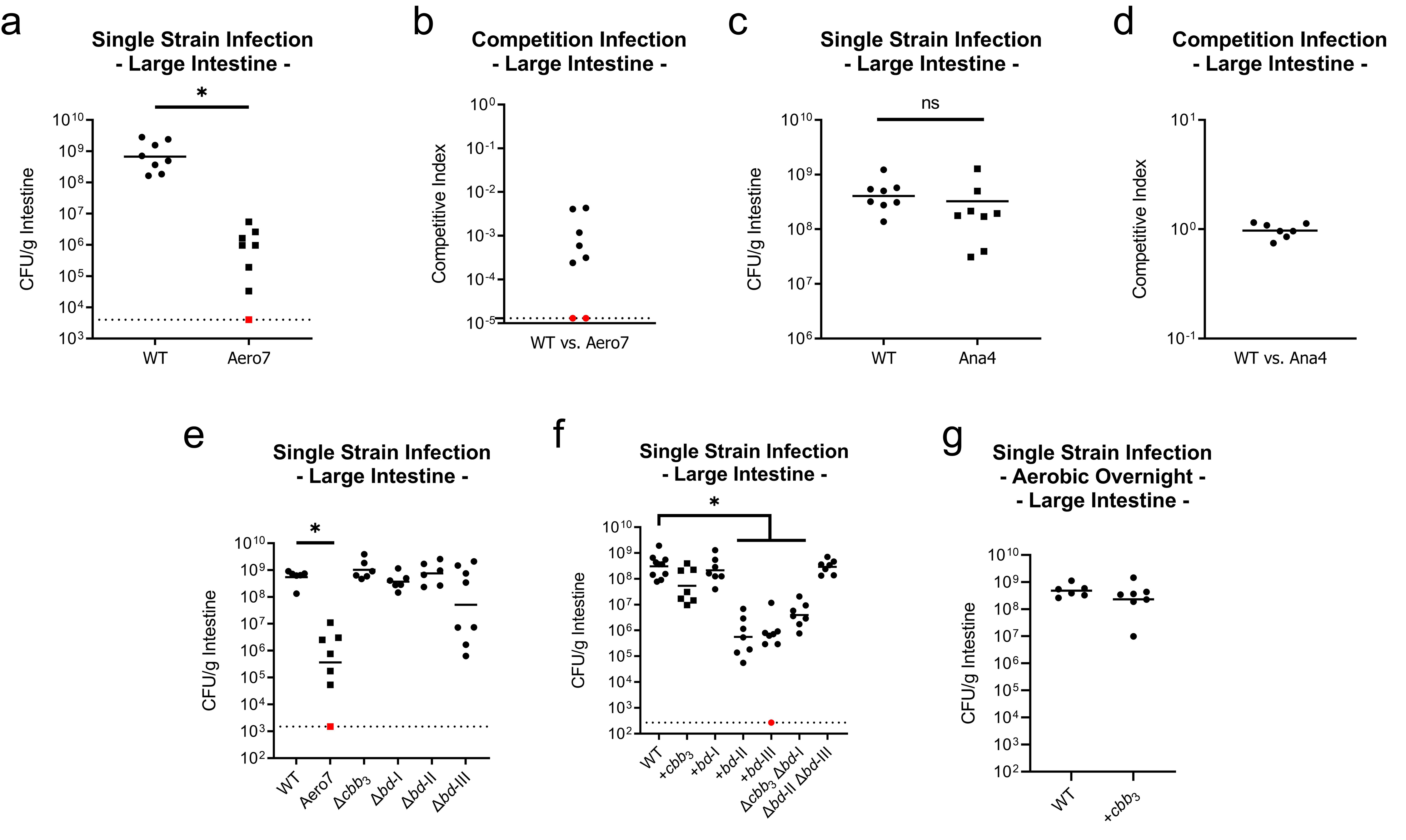

Supplement: S6 Fig — Aero7 and Ana4 colonization of the large intestine in both single strain and competition infections. (a) Single strain infection of strain Aero7. (b) Competition infection of strain Aero7. (c) Single strain infection of strain Ana4. (d) Competition infection of strain Ana4. Bars represent the geometric mean. Horizontal dashed lines indicate the limit of detection (LOD) and red dots indicate recovered CFUs were below the LOD. Competitive index scores were calculated as [(MutantOutput/WTOutput) / (MutantInput/WTInput)]. Statistical analysis was performed using GraphPad PRISM. *, P < 0.05. A Mann-Whitney U-test was used in the determination of significance between WT and Aero7. A Student’s t test was performed on log transformed data in the determination of significance between WT and Ana4. Single strain in vivo colonization assays in the large intestine for (e) individual and (f) combinatorial oxidase deletion strains. (g) Single strain colonization of aerobically prepared wild type and +cbb3 oxidase inoculums in the large intestine. Triple deletion mutant strains have a ‘+’ with an oxidase name (e.g. +cbb3), indicating the sole remaining oxidase, with the other three oxidases disrupted by mutation. Bars represent the geometric mean. Horizontal dashed lines indicate LOD, and red dots indicate recovered CFUs were below the LOD. Statistical analysis was performed using GraphPad PRISM. *, P < 0.05. A Mann-Whitney U-test was used in the determination of significance between WT and Aero7 and WT and +bd-III whereas an Analysis of Variance with post hoc Dunnett’s multiple comparisons test was conducted on log transformed CFU/g intestine for all other strain comparisons. (TIF) [file ppat.1010102.s007.tif]

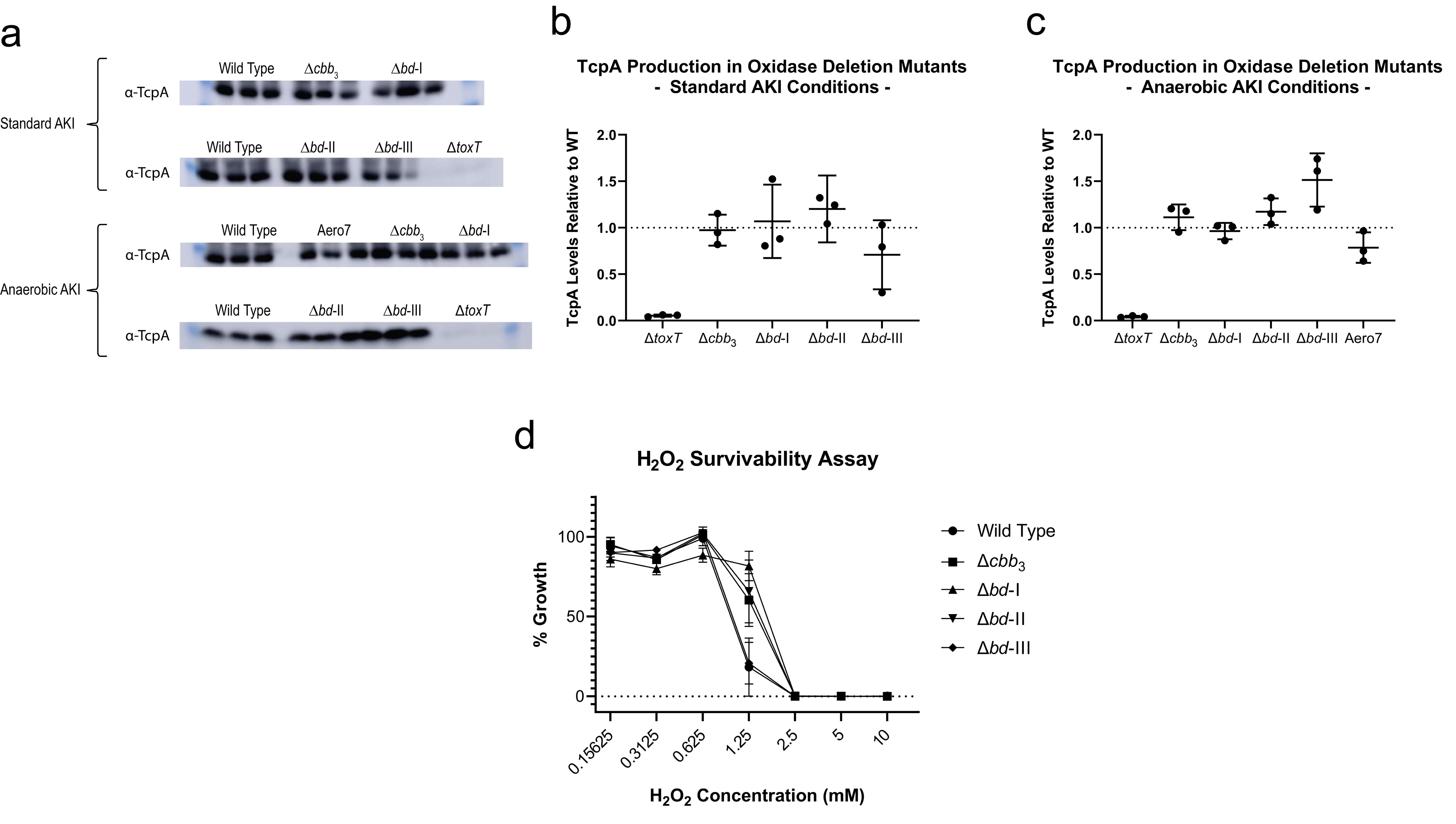

Supplement: S7 Fig — (a) Western blot visualization of TcpA, a required virulence factor in V. cholerae pathogenesis, in both standard and anaerobic AKI conditions. (b) Densitometry analysis of TcpA production in standard AKI conditions. (c) Densitometry analysis of TcpP production in anaerobic AKI conditions. TcpA levels are displayed as relative to TcpA production in wild type cells. ImageJ was used to perform the densitometry analysis across three biological replicates. Horizontal bars represent the arithmetic mean where error bars represent the standard deviation of the mean. (d) Hydrogen peroxide minimum inhibitory concentration determination of individual oxidase deletion strains. Growth percentage was calculated as a function of optical density for test strains in various concentrations of H2O2 (0.15625mM, 0.3125mM, 0.625mM, 1.25mM, 2.5mM, 5mM, and 10mM) divided by the optical density for wild type V. cholerae grown in LB media without H2O2. All strains showed signs of growth reduction at 1.25mM H2O2 and were all entirely inhibited for growth at 2.5mM H2O2. Data points represent the arithmetic mean of three biological replicates with error bars representing the standard error of the mean. (TIF) [file ppat.1010102.s008.tif]

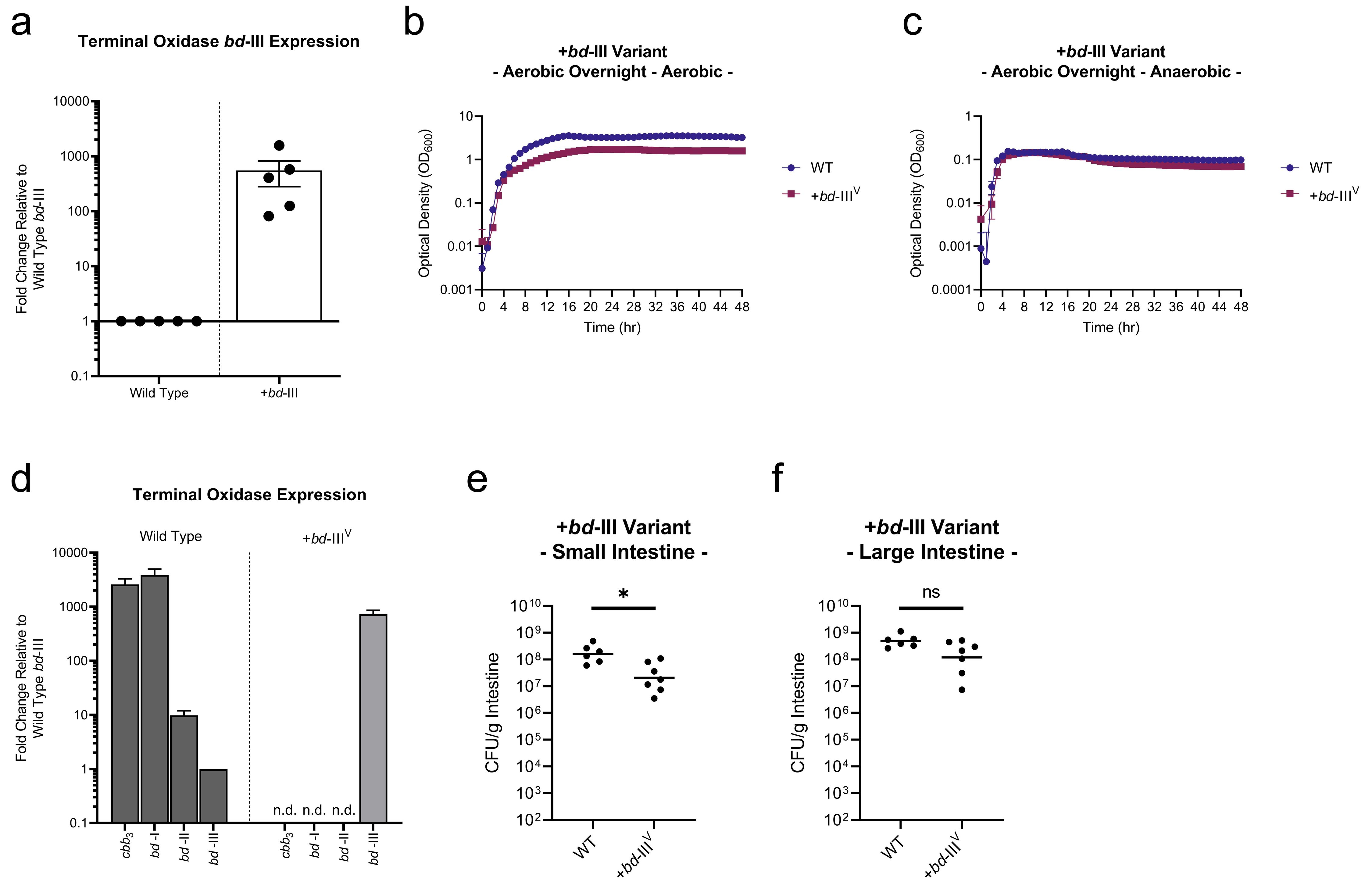

Supplement: S8 Fig — (a) Wild type and +bd-III strain (Δcbb3 Δbd-I Δbd-II) bd-III expression. Expression was determined for the primary subunit of the bd-III oxidase VC1571. Dots represent biological replicates of relative bd-III expression between +bd-III and wild type strains. Bars represent arithmetic mean with error bars representing the standard error of the mean. (b-c) Wild type and +bd-IIIV growth in LB. Inoculums were prepared aerobically and subsequently grown in aerobic and anaerobic conditions, respectively. Data points represent the mean of triplicate growth curves with error bars representing the standard error of the mean. (d) In vitro expression of terminal oxidases in +bd-IIIV strain. Expression was determined for the primary subunit of each oxidase complex (VC1442, VC1844, VCA0872, VC1571). Bars represent the arithemtic mean with error bars representing the standard error of the mean. (e) Single strain infection of +bd-IIIV in the small intestine. (f) Single strain infection of +bd-IIIV in the large intestine. Bars in single strain infections represent the geometric mean. Statistical analysis was performed using GraphPad PRISM. *, P < 0.05. A Student’s t test was performed on log transformed data in the determination of significance between WT and +bd-IIIV in both the small and large intestine. (TIF) [file ppat.1010102.s009.tif]
