# Supplementary material for: Vibrio cholerae requires oxidative respiration through the bd-I and cbb3 oxidases for intestinal proliferation
Source: PLoS Pathog. 2022 May 2;18(5):e1010102. doi: 10.1371/journal.ppat.1010102 (PMC9109917; doi:10.1371/journal.ppat.1010102)
Supplement: Attachment — Submitted filename: PLOSPathogens_Response to Reviewer Comments.docx [file ppat.1010102.s015.docx]

Response to reviewer comments - PPATHOGENS-D-21-02282

**Part I - Summary**
**Please use this section to discuss strengths/weaknesses of study, novelty/significance, general execution and scholarship.**

Reviewer #1: This paper reports the role of terminal oxidase in the survival and colonization abilities of V. cholerae. The authors analyzed the roles of cytochromes cbb3 and the bd and concluded that bd-I is the main oxidase in aerobic growth. They also found that these enzymes play important roles during colonization.

Some clarifications:

The authors should include data showing the growth rates of the different mutants

***Response: We have included new data showing growth of the different mutants in larger batch culture experiments. The relative growth rates of different mutants and wild type are clear from those experiments.***

The authors refer to the terminal oxidases of Pseudomonas aeruginosa. In P. aeruginosa, cytochrome aa3 which the most energy efficient enzyme is not the main oxidase expressed during aerobic and it is only expressed in very specific conditions. (starvation)
It is possible that V. cholerae uses bd-I as the “main oxidase” since the turnover of this enzyme is faster than that of cbb3.

The authors used a plate colorimetric assay to detect activity of cbb3. They need to make it clear that is a detection assay not a measurement.

***Response: We included text in the revised paper to address this point:***

***“This colorimetric detection assay indicates active cytochrome c oxidases but does not provide quantitative measurements of complex activity.”***

Overall, this paper offer important insights of the role of energy metabolism during the life cycle of V. cholerae.

***We thank the reviewer for this assessment and appreciate your comments on the manuscript.***

Reviewer #2: In this study, Andrew et al. investigate the function of multiple terminal oxidoreductases for aerobic and anaerobic respirations in Vibrio cholerae by using multiple knockout mutant strains. One of three bd-type quinol oxidases, bd-I, was identified to be the primary enzyme for aerobic growth and virulence to infant mouse. Anaerobic respiration was not important for infection in infant mouse.
The authors performed laborious work for construction of multiple mutants, competitive experiments, and infection assays. The obtained data seems to be plausible and important.

***Thank you for your conclusion about our work.***

Reviewer #3: The authors discovered that two of the four oxygen-dependent respiration mechanisms are essential for V. cholerae to grow during infection. Especially, they constructed the V. cholerae strains lacking aerobic or anaerobic respiration using a technique called MuGENT. In addition, using CoMPAS, they showed that bd-1 and cbb3 oxidases are essential for colonization in the infant mouse.

**Part II – Major Issues: Key Experiments Required for Acceptance**
Please use this section to detail the key new experiments or modifications of existing experiments that should be absolutely required to validate study conclusions.

Generally, there should be no more than 3 such required experiments or major modifications for a "Major Revision" recommendation. If more than 3 experiments are necessary to validate the study conclusions, then you are encouraged to recommend "Reject".

Reviewer #1: NO Major issues.

***Thank you***

Reviewer #2: I have a concern about the aerobic culture conditions. The authors used 96-well plates for the aerobic growth assays, but oxygen supply might not be enough in the culture.
I recommend confirming the results under highly aerating conditions using shaking flask or jar fermenter.

***Response: Thank you; to alleviate the above concern, we grew our individual and triple deletion strains in flask cultures where we observed a similar pattern in growth for all strains; although we have not included the data in this revised submission we present it here for review.***

**(Compare to Fig 2A and Fig3A)**

Reviewer #3: Here are some comments and suggestions to improve the manuscript;
1. In this manuscript, as their title, the authors asserted that the oxygen dependent respiration is essential for V. cholerae pathogenicity and proliferation in vivo. Thus, they showed the colonization abilities of mutants in infant mouse intestinal tract. But they did not show any pathogenicity of mutants. For example, were any mice dead within 20h after the infection? Please present the survival rate of them. Or present data to confirm the pathogenicity of them.

***Response:* *With respect to pathogenicity of V. cholerae, the infant mouse model as we typically carry it out, with a relatively low dose of infection, does not elicit signs of frank disease. A primary virulence factor of V. cholerae in both mice and humans is the toxin-coregulated pilus (TCP), required for colonization and expressed coordinately with the toxin. Thus we are using colonization as a proxy for pathogenicity. TCP expression is coordinated transcriptionally with the cholera toxin by the same transcription regulators ToxRS/TcpPH. This is the basis of wild type colonization in this model. Our respiratory pathway mutants are not defective for production of these virulence factors, so the lack of colonization is not due to lack of TCP expression, but instead – we conclude – due to inability to reach high levels of growth due to the respiratory defects. It is highly likely were we to use a mouse model where wild type infection led to poor survival the conclusions would be the same. Thus, we do not see the need to use a lot of mice to demonstrate something that will not change our conclusions. This was discussed with the Editor, Dr. Salama, who agreed with our viewpoint on this. We did change the title of the manuscript in response to this issue, however. The new title is: “*Vibrio cholerae requires oxidative respiration through the bd-I and cbb3 oxidases for intestinal proliferation”**

2. The authors determined that the bd-1 and cbb3 are essential for colonizing the small intestine of infant mouse. It would be informative to show how well these two gene clusters are conserved in V. cholerae genomes.

***Response: We analyzed the relatedness of the oxidase genes across Vibrionaceae and included this as Table S5. This is presented in the Discussion along with this text:***

***“We find high conservation (99%-100%) among V. cholerae strains for all oxidases (S5 Table). Looking at closely related Vibrionaceae, we find divergent genomic sequences for the cbb3 and bd-I oxidases and complete absence of the bd-II and bd-III oxidases complexes. This suggests that the cbb3 and bd-I oxidases are widely used to support aerobic growth among Vibrionaceae, but bd-II and bd-III are unique to the V. cholerae lineage, and the evolutionary rationale for their presence is not yet understood.”***

3. Page 9, line 205-207. Authors concluded from the growth assay and the expression profiles that the bd-1 oxidase is critical for aerobic respiration in V. cholerae as it transits from an anaerobic to aerobic environment. However, in Figure 2G, authors presented the expression levels of terminal oxidase genes in aerobic, microaerobic, and anaerobic conditions which were not conducted in transiting manner from one to another as they mentioned. To make the demonstration more plausible, please present the gradients in expression levels of terminal oxidase genes under transition condition from an anaerobic to aerobic environment.

***Response: The reviewer is correct in pointing out that our analysis of relative expression of the oxidases was carried out in discrete growth conditions. We clarify this in the revised manuscript. We suggest a requirement for the bd-I oxidase in transition from anaerobic to aerobic conditions based on growth curve assays in which bd-I was prepared anaerobically versus aerobically showing a reduced lag phase for aerobically prepare cultures (Figs 2a & 2e).***

***“All strains with a functional bd-I oxidase grew comparable to wild type during the shift from anaerobic to aerobic conditions whereas Δbd-I exhibited an extended lag phase that was ameliorated when inocula were prepared aerobically. The bd-I oxidase was the most abundantly expressed oxidase in anaerobic conditions, suggesting that accumulation of this oxidase in anaerobiosis readies V. cholerae for rapid growth during transition to aerobic atmospheric conditions. We hypothesize that expression and/or activity of the cbb_3_ oxidase is delayed during this transition period as bd-I deficient V. cholerae gradually exit lag phase, increasing cbb_3_ transcript abundance and exhibiting cbb_3_ activity in oxidative environments (S2B Fig)”***

4. In Figure 2 C and D. From in vitro competition assay, they demonstrated a competitive growth defect of both cbb3 and bd-1 deficient strains in aerobic conditions. In a competition assay, please display each CFU of the wild-type and the mutants as controls by culturing them individually.

***Response: We have counted CFU for each strain at the 20h timepoint and report these values in [New* Supplemental Figure 4A-B]. We discuss the likely possibility that due to the extended lag phase of a Δbd-I strain that WT V. cholerae is rapidly depleting nutrients preventing a late surge of Δbd-I which we see eventually occur in the growth curve of Fig 2a when cultured alone.***

5. Did you use the anaerobic chamber for all the anaerobic experiments? In line 597, 618 and 670, it says that deoxygenated LB was used for anaerobic conditions. Please clarify whether the anaerobic culture conditions of the assay were all referring to the experiment inside the anaerobic chamber.

***Response: Thank you for raising this point. We included a sentence in the Materials and Methods “Bacterial Strains and Growth Conditions” section indicating that deoxygenated LB was prepared by placing media in the Coy anaerobic chamber, allowing it to equilibrate with the anaerobic atmosphere prior to inoculation.***

***“Deoxygenated LB media used in indicated growth assays was prepared by setting loose capped media in the Coy anaerobic chamber for a duration of 16-24h.”***

6. In case of microaerobic culture, what does the term ‘microaerobic’ refer to? What are the standards and how did you set up the microaerobic condition? Please elaborate more about the culture conditions. If O2 concentration was set to certain degree in microaerobic culture, please state it.

***Response: Microaerobic conditions are now stated in the Materials and Methods “Bacterial Strains and Growth Conditions” section.***

***“For microaerobic growth conditions, an atmosphere of 5% oxygen, 10% carbon dioxide, 85% nitrogen was used.”***

7. In line 639. They checked the CFU of intestinal homogenates to test the colonizing abilities of aerobic or anaerobic mutants. For this assay, in which condition did you culture the plates of the two mutants? If you culture them in aerobic conditions, it is too favorable for one of the two mutants, which is making the experiment biased. It could be comprehensively explained with the CFU data from the suggestion no.4.

***Response: For clarification, we have included a sentence indicating that plates were consistently incubated in the anaerobic chamber to ensure growth of the oxidase deletion strains. This approach also had the added unintended benefit of not having to worry about colony size overgrowth when counting colonies.***

***“Plates were consistently incubated at 37°C in a Coy anaerobic chamber to ensure growth of oxidase deletion strains.”***

***Thank you for your reviews and suggestions to strengthen our manuscript.***

**Part III – Minor Issues: Editorial and Data Presentation Modifications**
Please use this section for editorial suggestions as well as relatively minor modifications of existing data that would enhance clarity.

Reviewer #1: The authors should include data showing the growth rates of the different mutants.

***Response: We have included new data showing growth of the different mutants in larger batch culture experiments. The relative growth rates of different mutants and wild type are clear from those experiments.***


The authors refer to the terminal oxidases of Pseudomonas aeruginosa. In P. aeruginosa, cytochrome aa3 which the most energy efficient enzyme is not the main oxidase expressed during aerobic and it is only expressed in very specific conditions. (starvation)
It is possible that V. cholerae uses bd-I as the “main oxidase” since the turnover of this enzyme is faster than that of cbb3.

***Response: We thank the reviewer for this insight.***

The authors used a plate colorimetric assay to detect activity of cbb3. They need to make it clear that is a detection assay not a measurement.

***Response: We included text in the revised paper to address this point:***

***“This colorimetric detection assay indicates active cytochrome c oxidases but does not provide quantitative measurements of complex activity.”***

Reviewer #2: There are two types in the bd-type quinol oxidases, i.e., the canonical bd-type and the cyanide-insensitive oxidase (CIO)-type. The CIO-type enzymes of Acetobacter and Pseudomonas species are reported to have low affinity to oxygen. bd-III of V. cholerae seems to be the CIO-type and might have high Km value. That might be the reason why the +bd-III grew faster when aerobic preculture was used (Fig. 3C).

***Response: We were interested in trying to discover what may be contributing to the +bd-III growth in aerobic conditions. We partially answered that through our work shown in Figure S8 from which it appears that elevated expression of +bd-III is supporting the recovered growth. Suppressor mutants rapidly emerge, one of which is a suppressor mutation in VC2301 which we touch on briefly in the discussion.***

Lines 79-80; The authors referred to oxygen reductases as terminal oxidase and reductases for alternative electron acceptors as terminal reductases. It might be better to define the terms “terminal oxidase” and “terminal reductase” here.

***Response: Thank you for this suggestion. We define these terms in the revised manuscript:***

***“Terminal oxidases are enzyme complexes that transfer electrons to oxygen in the final step of the electron transport chain. Terminal reductases perform the same function of electron transfer, however, electrons are transferred to substrates other than oxygen.”***

Lines 132, 136, and 164; Italicize “c” and “bc” of cytochromes c and bc1.
***Response: Thank you; we made these changes and similar adjustments were also made to S2 Fig legend.***

Figures 2, 3, and 4; Resolution of the graphs is very low, and it is hard to distinguish the lines.

***Response: Thank you. The files in our revised manuscript are provided as .tif at 600dpi resolution. The previous files were .jpg, which should not have affected quality greatly, as these were also 600dpi, and we apologize for that. Individual growth curve .tif files can also be provided for compilation by the journal.***

Lines 169-170; Indicate that the anaerobic growth is due to anaerobic fermentation.

***Response: This clarification has been made in the revised paper:***

***“All oxidase-disrupted mutants grew comparably to wild type in anaerobic conditions via anaerobic fermentation...”***

Lines 594-595; Shaking or aerating conditions for the aerobic cultivation should be indicated.

***Response: This clarification has been made in the revised paper:***

***“In aerobic conditions, four ml cultures were grown shaking at 210 rpm, 37°C whereas in anaerobic conditions four ml cultures were grown at 37°C static.”***

Sheet 2 of S3 Table and sheet 1 of S4 Table may be not related to this study.

***Response:* Thank you for catching this.**

Reviewer #3: 8. They stated ‘as the adult gut has greater anaerobic luminal volume compared to the limited luminal space in the infant’. Please add the reference.

***Response: We changed the text with a better description of our point (adding a reference), in response to the reviewer’s comment:***

***“This two-fold reduction observed in adult mice, and not infant, may be related to the myriad conditions that impact the overall oxygenation levels (or lack thereof) in the mammalian gut (40), and which may differ between infant and adult mice.”***
